# Expression changes of ribosomal proteins in phosphate- and iron-deficient Arabidopsis roots predict stress-specific alterations in ribosome composition

**DOI:** 10.1186/1471-2164-14-783

**Published:** 2013-11-13

**Authors:** Jinyan Wang, Ping Lan, Huimin Gao, Lu Zheng, Wenfeng Li, Wolfgang Schmidt

**Affiliations:** 1 State Key Laboratory of Soil and Sustainable Agriculture, Institute of Soil Science, Chinese Academy Sciences, Nanjing 210008, China; 2 College of Forest Resources and Environment, Nanjing Forestry University, Nanjing, China; 3 Institute of Plant and Microbial Biology, Academia Sinica, Taipei 11529, Taiwan

**Keywords:** Iron deficiency, Phosphate deficiency, Proteomics, Ribosomal proteins, RNA-seq, Translation

## Abstract

**Background:**

Ribosomes are essential ribonucleoprotein complexes that are engaged in translation and thus indispensable for growth. *Arabidopsis thaliana* ribosomes are composed of 80 distinct ribosomal proteins (RPs), each of which is encoded by two to seven highly similar paralogous genes. Little information is available on how RP genes respond to a shortage of essential mineral nutrients such as phosphate (Pi) or iron (Fe). In the present study, the expression of RP genes and the differential accumulation of RPs upon Pi or Fe deficiency in Arabidopsis roots were comprehensively analyzed.

**Results:**

Comparison of 3,106 Pi-responsive genes with 3,296 Fe-responsive genes revealed an overlap of 579 genes that were differentially expressed under both conditions in Arabidopsis roots. Gene ontology (GO) analysis revealed that these 579 genes were mainly associated with abiotic stress responses. Among the 247 RP genes retrieved from the TAIR10 release of the Arabidopsis genome (98 small subunit RP genes, 143 large subunit RP genes and six ribosome-related genes), seven RP genes were not detected in Arabidopsis roots by RNA sequencing under control conditions. Transcripts from 20 and 100 RP genes showed low and medium abundance, respectively; 120 RP genes were highly expressed in Arabidopsis roots. As anticipated, gene ontology (GO) analysis indicated that most RP genes were related to translation and ribosome assembly, but some of the highly expressed RP genes were also involved in the responses to cold, UV-B, and salt stress. Only three RP genes derived from three ‘sets’ of paralogous genes were differentially expressed between Pi-sufficient and Pi-deficient roots, all of which were induced by Pi starvation. In Fe-deficient plants, 81 RP genes from 51 ’sets’ of paralagous RP genes were significantly down-regulated in response to Fe deficiency. The biological processes ’translation’ (GO: 0006412), ’ribosome biogenesis (GO: 0042254), and ’response to salt (GO: 0009651), cold (GO: 0009409), and UV-B stresses (GO: 0071493)’ were enriched in this subset of RP genes. At the protein level, 21 and two RPs accumulated differentially under Pi- and Fe-deficient conditions, respectively. Neither the differentially expressed RP genes nor the differentially expressed RPs showed any overlap between the two growth types.

**Conclusions:**

In the present study three and 81 differentially expressed RP genes were identified under Pi and Fe deficiency, respectively. At protein level, 21 and two RP proteins were differentially accumulated under Pi- and Fe-deficient conditions. Our study shows that the expression of paralogous genes encoding RPs was regulated in a stress-specific manner in Arabidopsis roots, presumably resulting in an altered composition of ribosomes and biased translation. These findings may aid in uncovering an unexplored mechanism by which plants adapt to changing environmental conditions.

## Background

Ribosomes are large ribonucleoprotein complexes which synthesize the vast majority of cellular peptides. The structure of ribosomes is essentially the same in prokaryotes and eukaryotes in that they are composed of ribosomal RNAs (rRNAs) and ribosomal proteins (RPs), organized in a large and a small subunit. However, ribosomes of eukaryotes exhibit greater structural diversity, reflecting the higher complexity of the molecular mechanism underlying translation in the latter group [1,2]. In *Arabidopsis thaliana*, cytoplasmic ribosomes are composed of four distinct rRNAs (the large subunit contains 26S, 5S and 5.8S rRNA, the small subunit contains 18S rRNA) and 80 distinct ribosomal proteins (32 proteins in the small subunit and 48 proteins in the large subunit) [3,4]. The 80 RPs are encoded by a total of 249 genes; thus, several paralogous genes encode the same RP [1,4]. These paralogous genes could be redundant or, as indicated for certain RP genes, may be involved in specific plant processes or developmental stages [5-10]. For example, among the four paralogous genes encoding RPL11, one gene was highly expressed in cotyledons, whereas the other three genes were preferentially expressed in roots, developing anthers and pollen [11]. In addition, it has been reported that a mutation in *RPL23aA* (one out of two paralogous gene encoding RPL23a) lead to impaired growth and developmental abnormalities, suggesting that *RPL23aA* plays an essential role in fitness traits. By contrast, knockdown of the closely related gene family member *RPL23aB* had little effect on the phenotype [7]. Similarly, genes encoding RPS15a were differentially expressed in Arabidopsis, with one paralogous gene being completely transcriptionally quiescent, while the other three were highly expressed in mitotically active regions (e.g. flowers and buds) [12].

Ribosomal proteins are essential for protein synthesis and, consequently, play an important role in metabolism, cell division, and growth. In addition to their housekeeping functions, the phenotypes resulting from mutations in several different RP genes provide strong evidence that RPs participate as regulatory components in developmental processes [13]. Generally, RP mutants share developmental abnormalities such as reduced shoot growth, reduced cell proliferation and increased nuclear ploidy in leaf cells [13-15]. For example, a semi-dominant mutation in *RPL27aC* affected multiple aspects of plant shoot development, including leaf patterning, inflorescence and floral meristem function, as well as seed set [16]. Silencing of *RPS10* disturbed the ratio between the small and large subunits of mitoribosomes, causing an excess of the latter [17]. Introducing RPS6 antisense and RPL23aA RNAi constructs resulted in an altered number of cotyledons [7,18]. Also, a dominant missense mutation in *RPL10A* suppressed stem-elongation in comparison with the wild type [19]. Less severe phenotypes were reported for mutations in other RP genes. *POINTED FIRST LEAF (PFL)* encodes the small subunit RPS18 [20]. *pfl* mutants showed changes in the shape of early vegetative leaves from the spatulate wild-type shape to a pointed, narrow shape. Mutations in the *PFL2 (RPS13)* gene caused a delay in the transition to flowering and the production of more vegetative leaves than in the wild type [21]. In addition to effects on plant development and growth, RPs also took part in the response to stress. Under UV-B stress, RPL10 genes were differentially regulated in a dosage- and time-dependent manner; while *RPL10C* was induced and *RPL10B* was down-regulated at high UV-B intensity, *RPL10A* was not responsive to UV-B [22]. Another study showed that specific RPs changed in abundance in response to sucrose feeding, implying that different RPs are incorporated into ribosomes depending on the growth condition [23]. Transcripts of *RPS15aF* increased following treatment with cytokinin 6-benzylaminopurine (BAP) and auxin indole acetic acid (IAA), while abscisic acid (ABA) treatment decreased transcript abundance. In addition, transcripts of *RPS15aA, RPS15aD* and RPS15aF showed increased abundance upon temperature and mechanical stress [12].

Phosphate (Pi) is an essential macronutrient for plants. In addition to its structural role in nucleic acids and cell membranes, Pi has important functions in lipid and energy metabolism. Limited bioavailability of Pi often restricts growth in natural and agricultural ecosystems. Due to intense use of P fertilizers in agriculture, Pi resources have become limited. Therefore, understanding how plants adapt to low Pi availability is of critical importance to generate Pi-efficient plants. As a coping mechanism to limited Pi supply, plants undergo dramatic changes in metabolism, physiology, hormonal balance and gene expression [24-26]. Similarly, iron (Fe) is essential for the survival and proliferation of virtually all organisms. Iron is required for many vital enzymatic reactions such as DNA replication, lipid metabolism, and nitrogen fixation. In plants, Fe deficiency is a common nutritional disorder worldwide, causing low yield and quality of crop plants. To acquire sufficient Fe while avoiding toxicity, plants tightly control uptake, utilization, and storage in response to environmental availability [27].

At present, most studies on plant RPs focus on the developmental effects of RP mutants. We recently showed that Fe deficiency alters the expression of several RPs, presumably resulting in biased translation [28]. In the present study, changes in global expression pattern of RP genes in Arabidopsis roots upon Pi and Fe deficiency were compared at the transcriptomic and proteomic level in order to gain insights into the regulation of the response to Pi or Fe deficiency. It is shown that the expression of RPs followed stress-specific pattern, revealing a novel layer of regulation that may be critical for the fitness of plants under nutrient shortage.

## Results and discussion

### Comprehensive expression analysis of ribosomal protein genes in Arabidopsis roots

**Figure 1:**
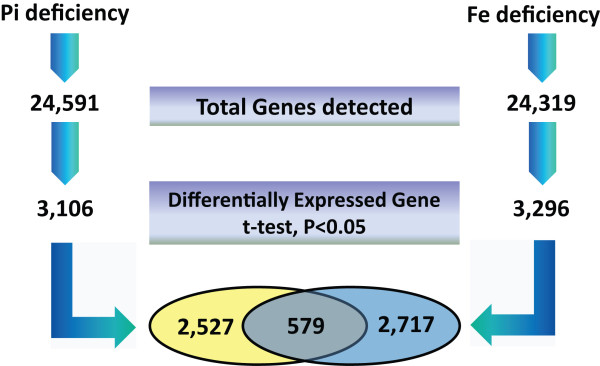
Transcriptional profile changes in Pi- and Fe deficient Arabidopsis roots.

The RNA-seq technology has proven to provide precise gene expression information [29] and accurate discrimination of differentially expressed genes under various conditions. Using this technology, we previously analyzed global gene expression changes upon Pi-deficiency and Fe-deficiency in Arabidopsis roots [30,31]. A total of 24,591 genes were detected in roots from Pi-deficient and Pi-sufficient plants by alignment to the TAIR10 annotation of the Arabidopsis genome (Figure 1). A set of 3,106 genes (13%) was differentially expressed under Pi deficiency (t-test, P value < 0.05, of which 1,497 genes were up-regulated and 1,609 genes were down-regulated). Using the same criteria, a total of 3,296 genes (14%, 1,462 induced genes and 1,834 depressed genes) were found to be differentially expressed between Fe-sufficient and Fe-deficient conditions among the 24,319 detected genes. Thus, a comparable number of genes were affected in both growth types. A subset of 579 genes was differentially expressed under both conditions.

**Figure 2:**
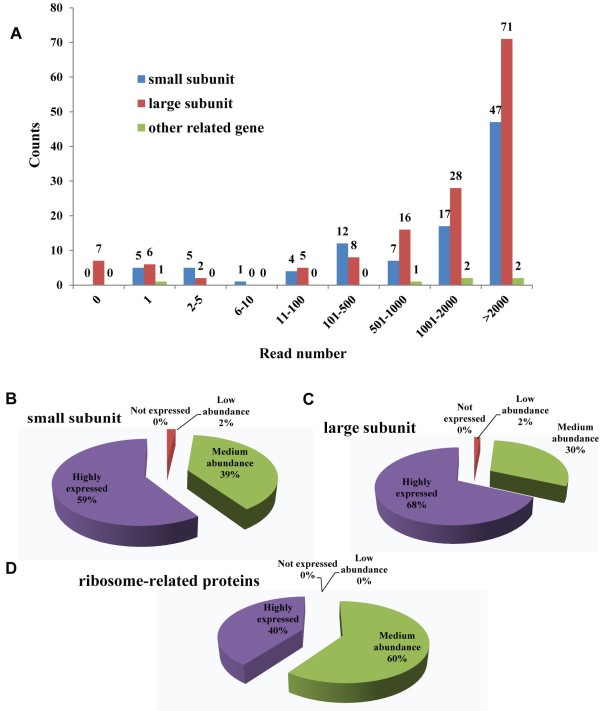
**Expression level of RP genes and proteins in Arabidopsis roots.** If the unique read number was zero in all three biological repeats under normal (Pi-/Fe-sufficient) conditions, the gene was defined as not expressed; low abundance of a transcript was defined by a unique read number <10 in either of three biological repeats; medium abundance was defined as read number between 11 and 2000, and highly expressed genes had more than 2000 reads in either of three biological repeats. **(A)** Read counts and gene numbers, **(B)** Percentage of the small subunit of ribosomal proteins identified in Arabidopsis roots. **(C)** Percentage of the large subunit of ribosomal proteins **(D)** Percentage of the ribosomal-related proteins.

Ribosomal proteins play critical roles in ribosome biogenesis, translation and post-translational modifications of proteins. A complete sequence data set for the 80 known RP families was compiled from the TAIR10 release of the Arabidopsis genome (http://www.arabidopsis.org/browse/genefamily/athr.jsp) [4,32], This data set (n = 256, Additional file 1) comprises all Arabidopsis paralogous genes associated with any specific RP family and other ribosome-related genes including eIF6 (eukaryotic Translation Initiation Factor 6), RACK1 (Receptor of Activated C Kinase) [33] and FER3 (FERRITIN 3) [34], which was shown to be tightly associated with ribosomes [5]. We defined the expression level of all RP genes in this data set according to an earlier study [35]. If the number of unique reads was zero in all three biological repeats under normal (Pi-/Fe-sufficient) condition, the gene was defined as not expressed; low abundance of a transcript was defined by a number of unique reads <10 in either of the three biological repeats; medium abundance was defined for genes with 11 to 2000 reads, and genes with more than 2000 reads in either of the three repeats were defined as highly expressed. On the basis of these criteria, in Arabidopsis roots subjected to Pi deficiency seven RP genes were not detected, transcripts of 20 RP genes were lowly abundant, 100 RP genes showed medium transcript levels, and 120 RP genes were highly expressed (Figure 2A and Additional file 2). The average read number of RP genes was 2,803; while on average 456 reads were associated with non-RP transcripts, indicating that RP paralogs were highly expressed in Arabidopsis roots. Previous data suggested that RP genes are retained in the Arabidopsis genome at higher levels than other types of gene [36], suggesting a high biological importance of this plant-specific diversity of RP genes. In growing yeast cells, 2000 ribosomes were produced per minute, and transcription of ribosomal RNA and RP genes account for 60% of total transcription [37]. The synthesis of RPs, ribosome assembly factors, and extra-ribosomal components involved in translation must simultaneously satisfy the plant’s demand for metabolism and growth, comprising an important component of the total cellular translational activity.

In many cases, different RP paralogs from the same RP families displayed differential expression. For example, among three paralogs of the RPL18 family, *RPL18B* and *RPL18C* were highly expressed with more than 5,000 reads, while the third member of this family, *RPL18A*, was not expressed. By analyzing public transcriptomic data (Genevestigator, https://www.genevestigator.com/gv/plant.jsp) [38], we found that expression of *RPL18A* is particularly low among all tissues and treatments, whereas *RPL18B* and *RPL18C* are highly expressed in some tissues. This suggests that *RPL18A* may be not functional or of minor importance, although retained in the genome during evolution. Pronounced differences were also observed for the expression of genes encoding the proteins of the large subunits L4, L22, L27a, L30, L37a, and L41. In a previous report, *RPS15aA*, *RPS15aD* and *RPS15aF*, but not *RPS15aC* transcripts were detected in all mature tissues by semi-quantitative RT-PCR [12]. Taken together, the differential expression of genes encoding RPs within specific ribosomal gene families indicates extensive ribosome heterogeneity at the intercellular level [6].

Within the 13 RP gene families with high read numbers (>2000) in Arabidopsis roots were six small (S3, S3a, S5, S6, S13, and S24) and seven large (L6, L7a, L10a, L15, L17, L24, and L38) ribosomal subunit proteins (Additional file 2). The highest expression level was observed for S3a, which was detected with an average read number of 6,917 for each paralog. High steady state levels of some RP gene transcripts may be indicative of fundamental roles of the encoded proteins in plant growth and development.

At the protein level, for 241 RP genes and 6 ribosome-related genes a total of 125 cognate proteins, accounting for nearly 50% of the detected genes, were identified in a proteomic approach ([30] and Additional file 3). This accounts for 75% of the large subunit proteins, 87.5% of the small subunit proteins, and 100% of the ribosome-related proteins. These results suggest that some RP families are necessary for translation in Arabidopsis roots, but not all of the paralogous proteins within one RP family take part in this process. Our data further demonstrate that RPs were more likely to be detected among the highly expressed genes than in genes with transcripts of medium or low abundance (Figure 2B, 2C and 2D). Notably, 41 highly expressed RP genes had no cognate peptides, supporting the observation that the gene expression level is not always correlated with protein abundance [30,35]. Many RP paralogs might have been detected, but without unique matches due to their high sequence identity. For example, for three paralogous proteins of the RPL6 family, RPL6A (AT1G18540), RPL6B (AT1G74060) and RPL6C (AT1G74050), 21 matching peptides have been detected, but only for RPL6A seven unique peptides were found, defining RPL6B and RPL6C as not present at the protein level although their corresponding transcript are high.

**Figure 3:**
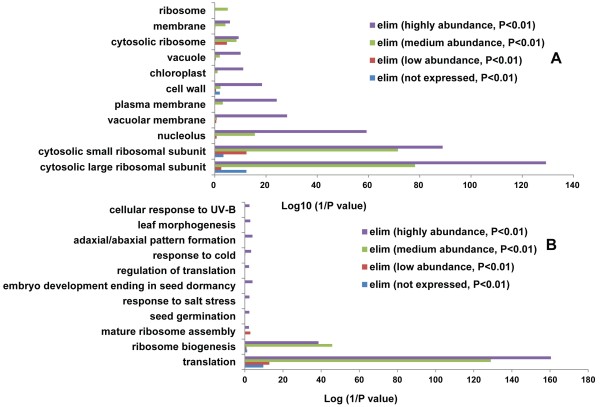
**Gene ontology (GO) enrichment analysis of the four types of RP genes in Arabidopsis roots. (A)** Subcellular location of the four types of RP genes. **(B)** Biological process of the four types of RP genes.

Gene Ontology (GO) enrichment analysis revealed that the products of most of the RP genes were localized in the cytosolic small and large ribosomal subunits, especially proteins encoded by medium and highly expressed genes (Figure 3A, P < 0.01). In addition, some biological processes such as ’cellular response to UV-B’ (GO: 0071493), ’response to cold’ (GO: 0009409), and ’response to salt stress’ (GO: 0009651) were overrepresented among the highly expressed genes (Figure 3B), indicating that RPs may also participate in the response to abiotic stresses. In addition, the GO categories ’seed germination’ (GO: 0009845), ’embryo development ending in seed dormancy’ (GO: 0009793), and ’leaf morphogenesis’ (GO: 0009965) were overrepresented in this group of RP genes (Additional file 4). In previous studies, mutations in some RP genes (i.e. *RPS5B, RPS6B, RPS11A, RPL3A, RPL8A, RPL19A, RPL23A,* and *RPL40B*) caused an embryo-lethal phenotype, [13,39,40]; thus, shortage in specific RPs could lead to severe growth defects. Mutations in the RP genes S13 and S18 were reported to cause abnormal leaf development [20,21]. Changes in leaf morphology in RP gene mutants might be caused by a diminished capacity to maintain protein synthesis at a level sufficient for proliferative cell divisions in the vasculature and palisade mesophyll, and for cell division progression at the leaf margin [13].

### Expression of RP genes in Pi-deficient Arabidopsis roots

Out of the 241 RP genes and six ribosomal-related genes annotated in TAIR10, only three were differentially expressed between Pi-sufficient and Pi-deficient Arabidopsis roots (t-test, P < 0.05; Table 1), all of which were up-regulated. Among the three genes, induction of *At1g74270* (*RPL35aC*) was more pronounced when compared with the other two genes, *AT3G04230* (*RPS16B*) and *AT3G02190* (*RPL39B*). In rice flowers, *RPL35a* was highly expressed under high temperature after the flowering stage [41], suggesting that this RP family is not only involved in Pi deficiency but may also play a role in heat stress response. In a previous report, transcript levels of *RPS16, RPL30* and *RPL13A* from Lupine cotyledons were found to be increased during cytokinin treatment relative to a 0 h water control [42]. Expression of *RPS16* was decreased after 5 h of incubation with ABA, indicating that RPS16 may have different functions in abiotic stresses.

**Table 1:** Differentially expressed RP genes in roots of Pi-deficient plants

**Gene ID**	**Protein name**	**Gene name**	**Type**	**Mean ± SD (-Pi/+Pi; P < 0.05)**
*AT3G04230*	S16	*RPS16B*	Small subunit	1.210 ± 0.085
*AT1G74270*	L35a	*RPL35aC*	Large subunit	1.425 ± 0.206
*AT3G02190*	L39	*RPL39B*	Large subunit	1.224 ± 0.086

The rapid accumulation of genome-wide gene expression data allows the creation of co-expression networks by examining the pair-wise relationship of genes over a large number of experimental conditions. In the co-expression network, a node is represented by a gene, and an edge is drawn between gene A and B if the correlation coefficient between these two genes is above a defined threshold [43]. Genes with similar expression pattern may be involved in the same biological process and potential functions of the unknown genes in a co-expression network can be predicted. [44,45]. In an attempt to identify Pi-responsive non-RP genes that are co-regulated with RP genes, we analyzed the correlation of the expression of the 3,106 differentially expressed genes including three RP genes. The results show that only one RP genes (*AT3G04230*) interacted with the 2-oxoglutarate (2OG) and Fe (II)-dependent oxygenase superfamily protein *AT3G13610* with a Pearson correlation coefficient (CF) cutoff of 0.7. With this CF threshold decreased to 0.5, all three differentially expressed RP genes and 262 other differentially expressed genes including some transcription factors and protein kinases could form a larger co-expression network (Additional file 5); and these genes were involved in diverse biological processes (Additional file 5).

At the protein level, 21 RPs accumulated differentially upon Pi deficiency (Table 2). Among them, only RPL12B (AT3G53430) was up regulated, all other proteins showed decreased abundance. Interestingly, among the three paralogous proteins in the RPL12 family, only RPL12B was induced, while the abundance of the other two RPL12 members was unchanged under Pi-deficient conditions. This suggests that RPL12B may play an important role in the Pi-stress response. To get a better understanding of the function of differentially expressed RPs, a protein-protein interaction network was constructed using the STRING database (http://string-db.org) [46] The network is presented in the ’evidence’ view, whereby lines connecting proteins represent the type of evidence (neighborhood, gene fusion, co-occurrence, co-expression, experiments, databases, text mining) used in predicting the association. All 21 differentially expressed RP proteins were clustered in a large protein-protein interaction module (Additional file 6).

**Table 2:** Differentially expressed ribosomal proteins in roots of Pi-deficient plants

**Protein ID**	**Protein name**	**Gene name**	**Type**	**Fold change**
AT5G20290	S8	*RPS8A*	Small subunit	0.738 ± 0.114
AT3G46040	S15a	*RPS15aD*	Small subunit	0.822 ± 0.039
AT2G09990	S16	*RPS16A*	Small subunit	0.810 ± 0.094
AT5G02960	S23	*RPS23B*	Small subunit	0.561 ± 0.166
AT4G39200	S25	*RPS25E*	Small subunit	0.689 ± 0.081
AT3G56340	S26	*RPS26C*	Small subunit	0.673 ± 0.186
AT1G18540	L6	*RPL6A*	Large subunit	0.831 ± 0.078
AT3G62870	L7a	*RPL7aB*	Large subunit	0.825 ± 0.032
AT2G18020	L8	*RPL8A*	Large subunit	0.777 ± 0.227
AT1G08360	L10a	*RPL10aA*	Large subunit	0.831 ± 0.068
AT3G53430	L12	*RPL12B*	Large subunit	1.385 ± 0.080
AT3G49010	L13	*RPL13B*	Large subunit	0.802 ± 0.155
AT4G27090	L14	*RPL14B*	Large subunit	0.815 ± 0.200
AT1G67430	L17	*RPL17B*	Large subunit	0.788 ± 0.185
AT3G49910	L26	*RPL26A*	Large subunit	0.744 ± 0.077
AT2G19730	L28	*RPL28A*	Large subunit	0.657 ± 0.236
AT1G77940	L30	*RPL30B*	Large subunit	0.832 ± 0.112
AT3G18740	L30	*RPL30C*	Large subunit	0.832 ± 0.107
AT4G18100	L32	*RPL32A*	Large subunit	0.768 ± 0.145
AT3G60245	L37a	*RPL37aC*	Large subunit	0.725 ± 0.114
AT2G43460	L38	*RPL38A*	Large subunit	0.772 ± 0.127

Alternative splicing (AS) is a mechanism that increases transcriptome and proteome complexity and controls developmental programs and responses to the environment in higher eukaryotes [47,48]. Previously, genome-wide differential AS features induced by Pi deficiency have been analyzed in Arabidopsis roots [31]. Further analysis revealed that a subset of 14 RP genes produced transcripts with retained introns; all of these intron retention events were induced by Pi shortage (i.e. more transcripts with retained introns were produced under Pi-deficient conditions; Additional file 7). Three out of the 14 encoded RPs were decreased in abundance under Pi deficiency. These results suggest that post-transcriptional regulatory processes such as alternative splicing may have affected the efficiency of translation [49].

### Expression of ribosomal protein genes in Fe-deficiency Arabidopsis roots

Using the criteria listed above, a total of 81 RP genes were differentially expressed in Fe-deficient Arabidopsis roots. In contrast to the up-regulated pattern of differentially expressed RP genes in roots of Pi-deficient plants, expression of all the 81 RP genes was decreased upon Fe deficiency. The RP gene with lowest relative expression level was the ribosome-associated protein gene *FERRITIN3* (*FER3; AT3G56090*), which was found in small polysome fractions isolated form the leaves of Arabidopsis plants in the dark [34]. FER3 was reported to be associated with the chloroplast and suggested to mediate coordination between the translation activity of cytosolic ribosomes and the function of chloroplasts in response to iron-based signals [50]. Thus, decreased expression levels of FER3 under Fe deficiency may be associated with the regulation of cellular Fe homeostasis.

**Figure 4:**
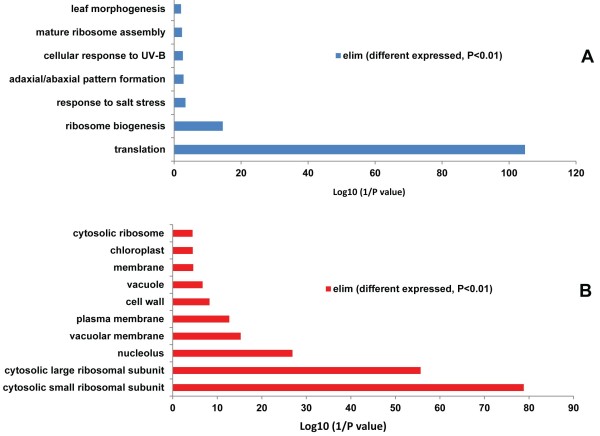
**Gene ontology (GO) enrichment analysis of Fe-responsive RP genes in Arabidopsis roots. (A)** Biological process of Fe-responsive RP genes. **(B)** Subcellular location of Fe-responsive RP genes.

Gene ontology analysis revealed that the 81 differentially expressed RP genes were chiefly involved in translation and ribosome biogenesis. In addition, the biological processes ’response to salt stress’, ’cellular response to UV-B’, and ’leaf morphogenesis’ were also enriched (Figure 4; Additional file 8). The 81 differentially expressed genes were derived from 23 small and 26 large ribosomal subunit RPs (Table 3), in which the transcript abundance of all paralogous genes from five RP families (L10a, L7a, S14, S3 and S5), was decreased. In the RPS15a family, three of the six paralogous genes (*RPS15aA, RPS15aB,* and *RPS15aD*) were down-regulated under Fe deficiency. These three genes showed increased transcript abundance following treatment with BAP, *RPS15aF* was also induced by IAA treatment. A similar trend of expression in paralogous genes with RPS15a family was observed under heat and cold stress [12]. It might thus be assumed that RPS15a is an important regulator involved in multiple abiotic and nutrient stresses.

**Table 3:** Differentially expressed RP genes in roots of Fe-deficient plants

**Gene ID**	**Protein name**	**Gene name**	**Type**	**Mean ± SD (-Fe/+Fe; P < 0.05)**
AT1G72370	Sa	*RPSaA*	Small subunit	0.851 ± 0.094
AT2G41840	S2	*RPS2C*	Small subunit	0.795 ± 0.057
AT2G31610	S3	*RPS3A*	Small subunit	0.832 ± 0.012
AT3G53870	S3	*RPS3B*	Small subunit	0.835 ± 0.051
AT5G35530	S3	*RPS3C*	Small subunit	0.831 ± 0.059
AT3G04840	S3a	*RPS3aA*	Small subunit	0.901 ± 0.046
AT4G34670	S3a	*RPS3aB*	Small subunit	0.893 ± 0.066
AT2G17360	S4	*RPS4A*	Small subunit	0.822 ± 0.083
AT5G07090	S4	*RPS4B*	Small subunit	0.836 ± 0.057
AT5G58420	S4	*RPS4D*	Small subunit	0.853 ± 0.057
AT2G37270	S5	*RPS5A*	Small subunit	0.844 ± 0.028
AT3G11940	S5	*RPS5B*	Small subunit	0.842 ± 0.065
AT5G59240	S8	*RPS8B*	Small subunit	0.717 ± 0.159
AT4G12160	S9	*RPS9A*	Small subunit	0.772 ± 0.053
AT5G15200	S9	*RPS9B*	Small subunit	0.862 ± 0.050
AT3G48930	S11	*RPS11A*	Small subunit	0.914 ± 0.027
AT1G15930	S12	*RPS12A*	Small subunit	0.782 ± 0.008
AT2G32060	S12	*RPS12C*	Small subunit	0.816 ± 0.080
AT3G60770	S13	*RPS13A*	Small subunit	0.809 ± 0.086
AT4G00100	S13	*RPS13B*	Small subunit	0.855 ± 0.003
AT2G36160	S14	*RPS14A*	Small subunit	0.775 ± 0.043
AT3G11510	S14	*RPS14B*	Small subunit	0.829 ± 0.050
AT3G52580	S14	*RPS14C*	Small subunit	0.786 ± 0.124
AT5G43640	S15	*RPS15E*	Small subunit	0.795 ± 0.063
AT1G07770	S15a	*RPS15aA*	Small subunit	0.900 ± 0.054
AT2G19720	S15a	*RPS15aB*	Small subunit	0.760 ± 0.040
AT3G46040	S15a	*RPS15aD*	Small subunit	0.831 ± 0.034
AT2G04390	S17	*RPS17A*	Small subunit	0.754 ± 0.016
AT2G05220	S17	*RPS17B*	Small subunit	0.758 ± 0.012
AT5G04800	S17	*RPS17D*	Small subunit	0.722 ± 0.019
AT1G22780	S18	*RPS18A*	Small subunit	0.831 ± 0.019
AT4G09800	S18	*RPS18C*	Small subunit	0.846 ± 0.028
AT3G45030	S20	*RPS20A*	Small subunit	0.785 ± 0.071
AT5G62300	S20	*RPS20C*	Small subunit	0.859 ± 0.042
AT5G02960	S23	*RPS23B*	Small subunit	0.878 ± 0.032
AT4G34555	S25	*RPS25D*	Small subunit	0.772 ± 0.082
AT4G39200	S25	*RPS25E*	Small subunit	0.844 ± 0.066
AT3G56340	S26	*RPS26C*	Small subunit	0.751 ± 0.058
AT5G47930	S27	*RPS27D*	Small subunit	0.884 ± 0.075
AT5G64140	S28	*RPS28C*	Small subunit	0.876 ± 0.033
AT3G44010	S29	*RPS29B*	Small subunit	0.860 ± 0.049
AT3G09200	P0	*RPP0B*	Large subunit	0.823 ± 0.103
AT1G01100	P1	*RPP1A*	Large subunit	0.757 ± 0.042
AT5G47700	P1	*RPP1C*	Large subunit	0.790 ± 0.049
AT1G43170	L3	*RPL3A*	Large subunit	0.801 ± 0.051
AT5G39740	L5	*RPL5B*	Large subunit	0.898 ± 0.042
AT5G40130	L5	*RPL5C*	Large subunit	0.934 ± 0.054
AT1G74060	L6	*RPL6B*	Large subunit	0.871 ± 0.067
AT2G44120	L7	*RPL7C*	Large subunit	0.818 ± 0.019
AT2G47610	L7a	*RPL7aA*	Large subunit	0.858 ± 0.020
AT3G62870	L7a	*RPL7aB*	Large subunit	0.843 ± 0.060
AT4G36130	L8	*RPL8C*	Large subunit	0.836 ± 0.081
AT1G33120	L9	*RPL9B*	Large subunit	0.843 ± 0.033
AT1G33140	L9	*RPL9C*	Large subunit	0.850 ± 0.018
AT1G14320	L10	*RPL10A*	Large subunit	0.900 ± 0.017
AT1G08360	L10a	*RPL10aA*	Large subunit	0.838 ± 0.066
AT2G27530	L10a	*RPL10aB*	Large subunit	0.817 ± 0.063
AT5G22440	L10a	*RPL10aC*	Large subunit	0.861 ± 0.030
AT2G42740	L11	*RPL11A*	Large subunit	0.793 ± 0.067
AT3G58700	L11	*RPL11B*	Large subunit	0.761 ± 0.040
AT5G23900	L13	*RPL13D*	Large subunit	0.860 ± 0.042
AT3G07110	L13a	*RPL13aA*	Large subunit	0.764 ± 0.009
AT3G24830	L13a	*RPL13aB*	Large subunit	0.731 ± 0.055
AT4G13170	L13a	*RPL13aC*	Large subunit	0.769 ± 0.066
AT4G16720	L15	*RPL15A*	Large subunit	0.810 ± 0.031
AT1G27400	L17	*RPL17A*	Large subunit	0.831 ± 0.073
AT5G27850	L18	*RPL18C*	Large subunit	0.901 ± 0.029
AT2G34480	L18a	*RPL18aB*	Large subunit	0.787 ± 0.024
AT3G14600	L18a	*RPL18aC*	Large subunit	0.852 ± 0.022
AT1G02780	L19	*RPL19A*	Large subunit	0.891 ± 0.040
AT1G57660	L21	*RPL21E*	Large subunit	0.822 ± 0.055
AT1G57860	L21	*RPL21G*	Large subunit	0.818 ± 0.045
AT3G22230	L27	*RPL27B*	Large subunit	0.808 ± 0.067
AT1G70600	L27a	*RPL27aC*	Large subunit	0.819 ± 0.027
AT2G19730	L28	*RPL28A*	Large subunit	0.872 ± 0.053
AT1G77940	L30	*RPL30B*	Large subunit	0.867 ± 0.036
AT5G46430	L32	*RPL32B*	Large subunit	0.901 ± 0.031
AT5G02610	L35	*RPL35D*	Large subunit	0.905 ± 0.036
AT1G18080	RACK1A	*RACK1A*	Other type gene	0.758 ± 0.031
AT1G48630	RACK1B	*RACK1B*	Other type gene	0.840 ± 0.055
AT3G56090	FER3	*FER3*	Other type gene	0.511 ± 0.074

Co-expression analysis of the 81 differentially expressed RP genes under Fe deficiency yielded a network consisting of 68 nodes and 1,827 edges (Additional file 9). This network could be further divided into one larger and one smaller clusters, the larger of which contained much more nodes (66/68) and edges, and every node (ribosomal protein gene) was correlated with other RP genes (27 edges per node). Similarly, with the aim of identifying other Fe-responsive non-RP genes that interact with RP genes, we put the 3,296 differentially expressed genes including 81 RP genes together to do the co-expression analysis. Results showed that a total of 77 RP genes and 221 Fe-responsive non-RP genes comprise one bigger and two smaller clusters with Pearson correlation coefficient cutoff of 0.7 (Additional file 10).

Proteomic profile analysis showed that only two RPs were differentially expressed between Fe-deficient and Fe-sufficient Arabidopsis roots [49]. The two proteins were from the RPL22 family and showed decreased abundance, although the transcriptional expression of these two genes remained unchanged (Table 4). Analysis of AS revealed three RP genes with differential alternative donor/acceptor splices sites and six RP genes with differential intron retention in roots of Fe-deficient plants (Additional file 11). None of these RP genes were transcriptionally or translationally Fe-responsive.

**Table 4:** Differentially expressed ribosomal proteins in roots of Fe-deficient plants

**Protein ID**	**Protein name**	**Gene name**	**Type**	**Fold change**
AT3G05560	RPL22	*RPL22B*	Large subunit	0.5 ± 0.22
AT5G27770	RPL22	*RPL22C*	Large subunit	0.58 ± 0.22

### Comparative expression pattern of RP genes under Pi and Fe deficiency

**Figure 5:**
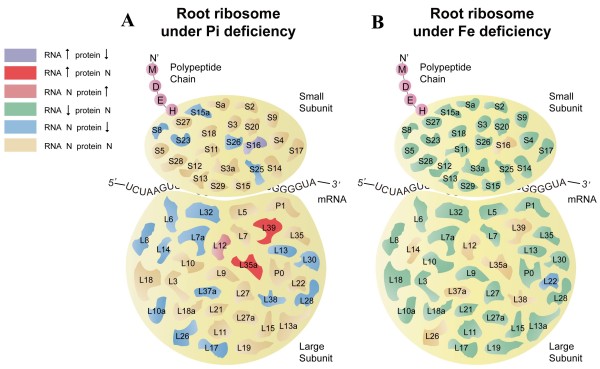
**Phosphate and Fe deficiency-induced mRNA and protein changes and predicted changes in ribosomal composition. (A)** Pi deficiency, **(B)** Fe deficiency. Purple shapes represent ribosomal protein families that were up-regulated at the RNA level and down-regulated at the protein level. Red shapes represent ribosomal protein families that were up-regulated at the RNA level and that were not detected at the protein level. Pink shapes represent ribosomal protein families not detected at the RNA level and up-regulated at the protein level. Green shapes represent ribosomal protein families down-regulated at the RNA level and not detected at the protein level. Blue shapes represent ribosomal protein families not detected at the RNA level and down-regulated at the protein level. Yellow shapes represent ribosomal protein families not detected at both RNA and protein level.

Comparison of the differentially expressed RP genes and RPs between Pi- and Fe- deficient Arabidopsis roots showed no overlap between the two growth types, suggesting that distinct RPs are involved in the responses of plants to Pi- and Fe-deficient. Interestingly, most RP genes encoding proteins of the large subunit were affected by Pi deficiency, while the majority of differentially RP genes encoding RPs under Fe deficiency were from both the small and the large subunit (Figure 5). Furthermore, the expression pattern of RPs upon Pi deficiency was more complex than that observed under Fe-deficient conditions. Nevertheless, one of the common observations for these two treatments is that there was a large discrepancy between the RP genes and PR proteins affected by Pi deficiency (compare Tables 1 and 2) as well as under Fe deficiency conditions (compare Tables 3 and 4). Our previous studies [35,49] have found that the concordance between differences in the abundance of mRNA and its encoded protein is not always high due to several factors: Firstly, proteins with low abundance might not be reliably detected and thus did not match the criteria that define them as differentially accumulated whereas the transcript levels of the corresponding genes are significantly changed. Secondly, several proteins with high abundance accumulated differentially upon Fe/Pi deficiency, but they have important housekeeping functions and the transcript levels were not changed. Finally, a change in transcript abundance may or may not be translated into changes in protein level; posttranscriptional regulatory processes such as alternative splicing, RNA processing, and other processes may affect the efficiency of translation. A generally low congruency of proteomic and transcriptional profiles has been reported previously [51].

## Conclusions

**Figure 6:**
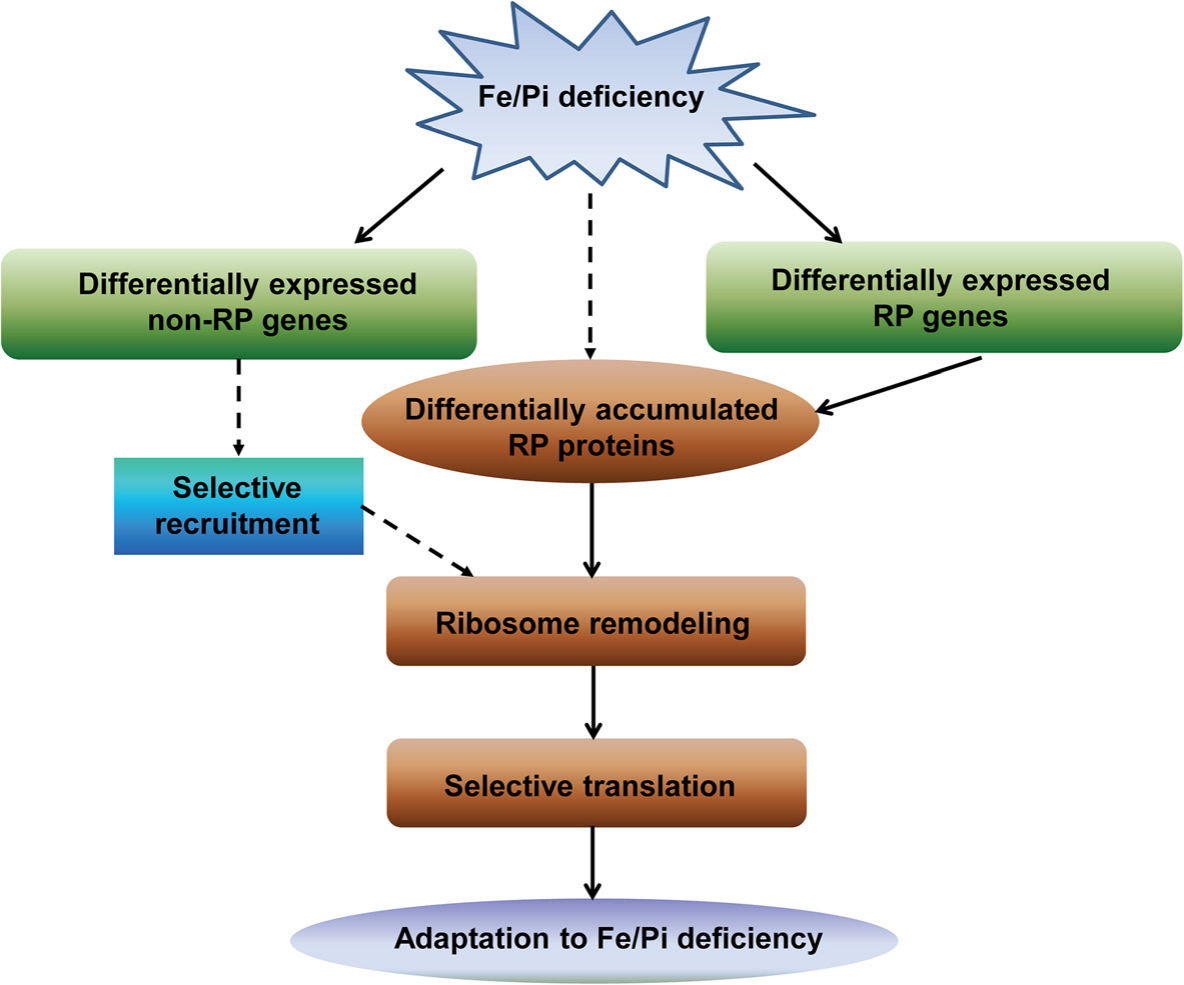
**A model of RP genes and proteins involved in Pi/Fe deficiency homeostasis.** Upon Pi/Fe deficiency (and other abiotic stresses), plants sense the stress signal and the RPs, RP genes, as well as non-RP genes are differentially expressed, inducing downstream signal transduction. Some genes might be selectively recruited to the remodeled ribosome leading to biased translation, probably adapting plants to Pi- and Fe-deficiency.

In summary, our results suggest that the composition of ribosomes is changed in a stress-specific manner as part of an adaptation to Pi and Fe deficiency (and might be remodeled in other abiotic stresses), probably biasing protein translation (Figure 6). Our findings further suggest that RPs are not only involved in protein translation but also in the response of plants to nutrient deficiency, possibly altering the composition of ribosomes in roots of Pi- or Fe-deficient plant. The functional divergence of RP paralogs could be attributable to genetically- or epigenetically-driven neofunctionalization and/or subfunctionalization, leading to highly specialized paralogs associated with Pi- and Fe-deficient processes. These findings may have important implications for understanding the molecular functions of RP genes in response to changing environmental conditions.

## Methods

### Plant growth

Arabidopsis (*Arabidopsis thaliana*) plants were grown in a growth chamber on an agar-based medium as described previously [52]. Seeds of the Col-0 accession were obtained from the Arabidopsis Biological Resource Center. Seeds were surface sterilized by immersing them in 5% (v/v) NaOCl for 5 min and 96% ethanol for 7 min, followed by four rinses in sterile water. Seeds were placed onto Petri dishes and kept for 1 d at 4°C in the dark; the plates were then transferred to a growth chamber and grown at 21°C under continuous illumination (50 μmol m-2 s-1; Philips TL lamps). The medium was composed of (mM): KNO3 (5), MgSO4 (2), Ca(NO3)2 (2), KH2PO4 (2.5); (μM): H3BO3 (70), MnCl2 (14), ZnSO4 (1), CuSO4 (0.5), NaCl (10), Na2MoO4 (0.2); and 40 μM Fe-EDTA solidified with 0.3% Phytagel (Sigma-Aldrich). Suc (43 mM) and 4.7 mM MES were included, and the pH was adjusted to 5.5. After 10 d of pre-cultivation, plants were transferred either to fresh agar medium without Fe and with 100 μM 3-(2-pyridyl)-5,6-diphenyl-1,2,4-triazine sulfonate, or medium without phosphate, or fresh control medium and grown for another 3 d. The lower concentration of potassium because of the absence of KH2PO4 in the phosphate-free medium was corrected by the addition of KCl.

### Data collection and data mining

Transcriptomic data of roots from 13-d-old Arabidopsis seedlings grown in the presence or absence of Pi or Fe were downloaded from the NCBI SRA database (Pi: SRA050356.1; Fe: SRA045009) and analyzed as described in [30]. The proteomic data of Pi- or Fe-deficient Arabidopsis roots from 13-d-old Arabidopsis seedlings were retrieved from [30] and [49], respectively. The cytosolic RP genes of *Arabidopsis thaliana* were compiled based on the Arabidopsis Genome Initiative (AGI) identification numbers provided by [4,32] (available in TAIR10).

### Gene ontology analysis

Enrichment analysis of GO categories was performed with the Gene Ontology Browing Utility (GOBU) toolbox [53], using the TopGo ’elim’ algorithm [54] for the aspects ’biological process’ and ’subcellular localization’. The selected categories were sorted from the lowest to the highest P value (P < 0.01).

### Generation of co-expression networks and modules of Fe-responsive ribosomal protein genes using the MACCU toolbox

To generate root-specific networks of Pi- and Fe-responsive ribosomal protein genes, differentially expressed RP genes in Arabidopsis roots were identified using a Student’s t-test at a P value < 0.05. Gene networks were constructed based on 300 publicly available root-related microarray experiments using the MACCU toolbox as described in [44], with a Pearson’s correlation threshold of 0.7. The generated co-expression networks were visualized by Cytoscape (http://www.cytoscape.org). If one gene cluster did not have any connection (edges) to any other cluster in the co-expression network, we referred to such a cluster as module.

## Competing interests

The authors declare that they have no competing interests.

## Authors’ contributions

JYW, PL, and WL performed the data analysis and drafted the manuscript. HMG, LZ, and WS participated in the analysis of the data. PL and WL conceived and supervised the study. All authors approved the final version of the manuscript.

## Supplementary Material

Additional file 1 List of RP genes and ribosome-related genes in the Arabidopsis genome. Click here for file

Additional file 2 Expression of 247 RP and ribosome-related genes in Arabidopsis roots. Click here for file

Additional file 3 125 RPs identified in Arabidopsis roots. Click here for file

Additional file 4 GO enrichment analysis of the 120 highly expressed RP genes in Arabidopsis roots. Click here for file

Additional file 5 Gene lists of differentially expressed non-RP genes involved in the co-expression networks under Pi deficiency. Click here for file

Additional file 6 Protein-protein interaction of 21 differentially expressed proteins under Pi deficiency in Arabidopsis roots. Click here for file

Additional file 7 Differentially alternative splicing (DAS) features under Pi deficiency. Click here for file

Additional file 8 GO enrichment analysis of the 81 differentially expressed RP genes under Fe deficiency. Click here for file

Additional file 9 Co-expression relationships of 81 differentially expressed genes under Fe deficiency in Arabidopsis roots. Click here for file

Additional file 10 Gene lists of differentially expressed non-RP genes involved in co-expression networks under Fe deficiency. Click here for file

Additional file 11 DAS features under Fe deficiency. Click here for file
