# Supplementary figures and images for: Expression changes of ribosomal proteins in phosphate- and iron-deficient Arabidopsis roots predict stress-specific alterations in ribosome composition
Source: BMC Genomics. 2013 Nov 13;14:783. doi: 10.1186/1471-2164-14-783 (PMC3830539; doi:10.1186/1471-2164-14-783)

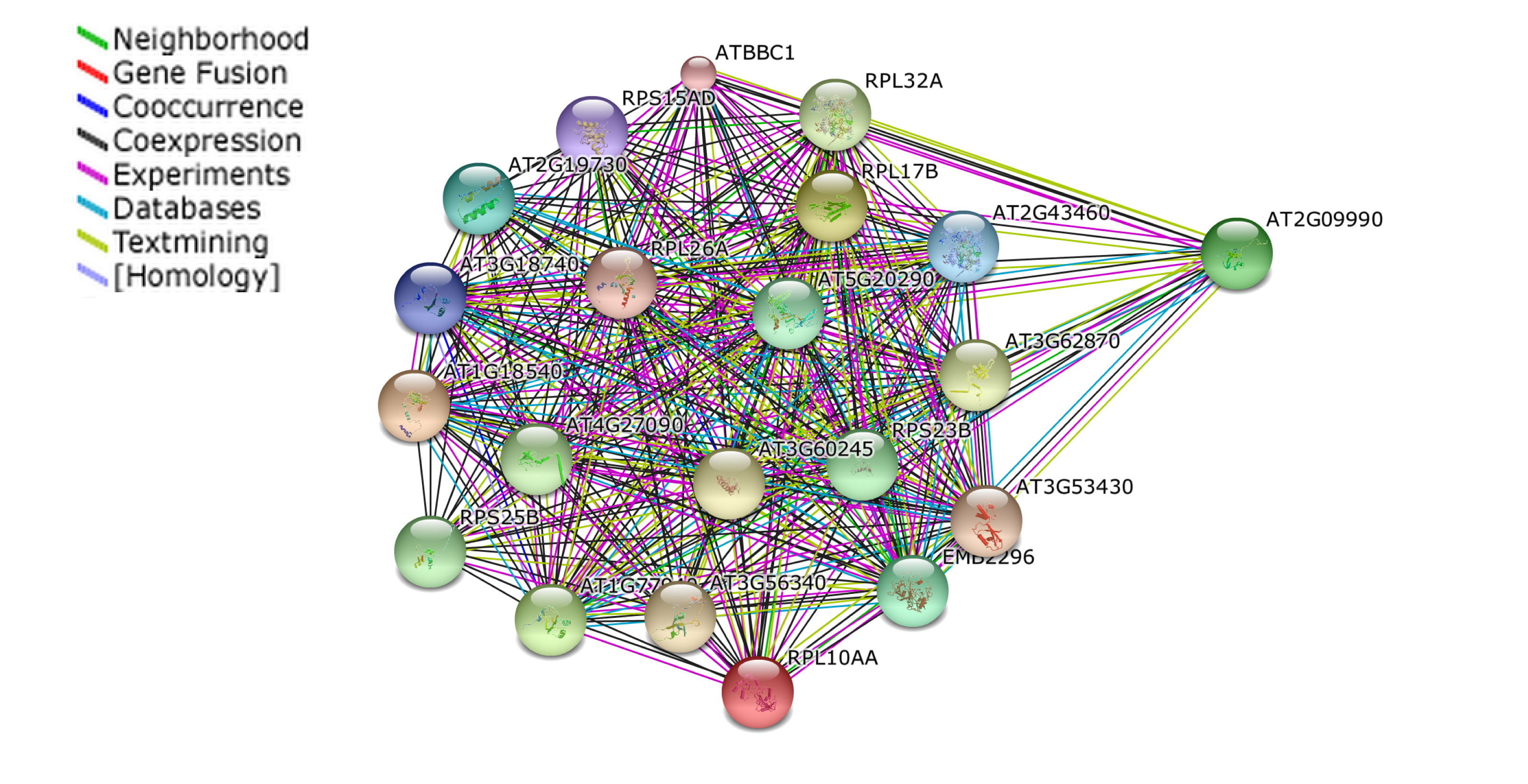

Supplement: Additional file 6 — Protein-protein interaction of 21 differentially expressed proteins under Pi deficiency in Arabidopsis roots. [file 1471-2164-14-783-S6.jpeg]

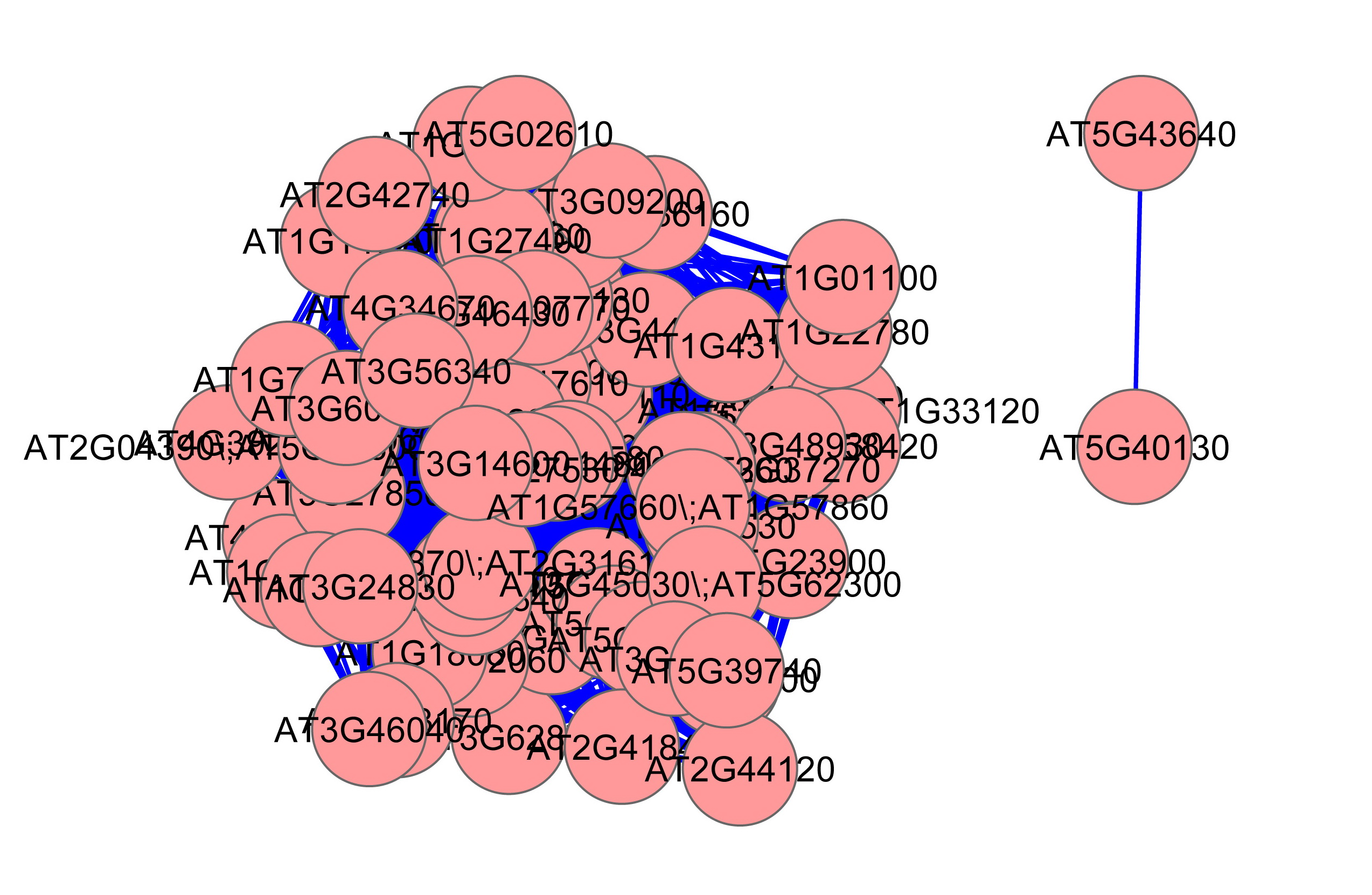

Supplement: Additional file 9 — Co-expression relationships of 81 differentially expressed genes under Fe deficiency in Arabidopsis roots. [file 1471-2164-14-783-S9.jpeg]
